# Effects of Alfalfa Fiber Meal on the In Vitro Fermentation Characteristics and Community Structure of the Colonic Microbiota of Sows

**DOI:** 10.3390/microorganisms14030548

**Published:** 2026-02-27

**Authors:** Shuhang Zhang, Ruicheng Yu, Congbin Chen, Zhichang Wang, Xiaoyan Zhu, Yalei Cui, Boshuai Liu, Yinghua Shi

**Affiliations:** 1 College of Animal Science and Technology, Henan Agricultural University, Zhengzhou 450046, China; 17716388770@163.com (S.Z.); 18836905164@163.com (R.Y.); 18638969619@163.com (C.C.); zcwang@henau.edu.cn (Z.W.); zxy_0512@163.com (X.Z.); yaleicui423@henau.edu.cn (Y.C.); 2 Henan Key Laboratory of Grassland Resources Innovation and Utilization, Zhengzhou 450046, China; 3 Henan Engineering Research Center for Grass Industry, Zhengzhou 450046, China

**Keywords:** alfalfa fiber meal, in vitro fermentation, pregnant sows, short-chain fatty acids, sow gut microbiota

## Abstract

The gut microbiota plays a critical role in hindgut health and nutrient utilization in monogastric animals. Alfalfa fiber meal (AFM) was rich in essential vitamins and minerals as a valuable nutritional supplement. In this study, an in vitro fermentation model was established using fecal microbiota from pregnant sows as the inoculum to evaluate the effects of different supplementation levels of alfalfa fiber powder (AFM) on fermentation metabolites and microbial community composition, with particular attention to interactions between the microbiota and metabolites. Fecal inocula from healthy sows were fermented with AFM at three inclusion levels: low (LAFM: 50 mg), medium (MAFM: 100 mg), and high (HAFM: 200 mg). Fermentation samples were collected at 8, 12, 24, and 36 h for analysis of gas production and short-chain fatty acid (SCFAs) concentrations. Microbial community composition was characterized at 36 h, followed by correlation analysis between dominant genera and fermentation parameters. The results showed that total gas and hydrogen production increased significantly with both AFM level and time, while hydrogen sulfide decreased across all treatments. Methane production rose in the early stages and remained elevated only in the high-AFM group. AFM supplementation promoted the production of total and individual short-chain fatty acids in a dose- and time-dependent manner. Microbial analysis revealed reduced *Fusobacterium* and increased *Lactobacillus*, *Bacteroides*, and *Collinsella*, with high AFM further enriching *Prevotella* and *Megasphaera*. Positive correlations were observed between SCFA production and *Collinsella*, *Prevotella*, and *Olsenella*, whereas hydrogen sulfide correlated negatively with *Prevotella* and *Sharpea*. AFM effectively improved gut microbial composition and fermentation efficiency, with 100 mg identified as a more balanced level of fermentation additive supplementation for pregnant sows under in vitro conditions.

## 1. Introduction

The animal gut constituted a complex ecosystem harboring vast microbial communities that exceeded host cell numbers in abundance and genetic diversity [1]. These gut microbiota engage in complex interactions with the host and play a crucial role in maintaining host health, whereas dysbiosis may trigger various pathological conditions, such as intestinal infections, inflammatory diseases, and metabolic disorders [2,3]. Consequently, modulation of the gut microbiota emerged as a significant research focus in both medical and agricultural sciences. In recent years, fecal microbiota transplantation (FMT) gained recognition as an innovative therapeutic approach for gut microbiota modulation, demonstrating promising clinical outcomes, particularly in treating recurrent intestinal infections [4]. The fundamental principle of FMT involved transferring functional microbial communities from healthy donors to restore ecological balance in recipients’ guts. However, the widespread application of FMT faced challenges related to donor variability and the standardization of microbial preparation protocols.

FMT is an “in vivo intervention for reconstructing the gut microbiota,” whereas in vitro fermentation is a “model for ex vivo evaluation of microbiota function and substrate fermentability”; the two are often used as complementary lines of evidence. In this context, in vitro fermentation technology offered a novel platform for large-scale, standardized cultivation of gut microbiota. This system enabled high-throughput microbial culture under controlled conditions that simulated the intestinal environment [5], providing not only consistent microbial sources for FMT but also an effective platform for investigating the effects of dietary components and pharmaceuticals on the gut microbiota. The nutritional composition of the culture medium represented a critical determinant of microbial growth and metabolism in such in vitro systems. Given the diversity and complex interactions of porcine colonic microbiota, establishing representative in vitro models would significantly advance research on pig gut microbial ecology.

With the global livestock industry entering the “post-antibiotic era,” identifying safe and effective antibiotic alternatives became an urgent priority. Dietary fiber represents an important nutritional component that can modulate gut microbiota through multiple mechanisms, thereby improving intestinal health, enhancing immune barriers, and boosting production performance [6]. Alfalfa fiber meal (AFM), obtained through stem–leaf separation technology, was rich in high-quality crude fiber and provided essential vitamins and minerals as a valuable nutritional supplement [7,8]. In feed formulations, fiber components helped balance energy-to-protein ratios, consequently improving animal growth performance and product quality [9].

The high costs associated with sow maintenance and the technical challenges in obtaining intestinal content samples made in vitro fermentation an ideal approach for investigating AFM’s effects on sow gut microbiota and their metabolic outputs. The high costs associated with sow maintenance and the technical challenges in obtaining intestinal content samples made in vitro fermentation an ideal approach for investigating AFM’s effects on sow gut microbiota and their metabolic outputs. This facilitates the rapid translation of research findings into practical applications while improving production efficiency. Although alfalfa fiber has been applied to some extent in animal production, the specific mechanisms by which it acts on the sow gut microbiota remain insufficiently understood. This study focuses on gestating sows because gestation is a distinct physiological stage closely related to production, during which the gut microbiota and fermentative metabolism may differ from those of non-pregnant animals. We hypothesized that AFM increases SCFAs and enriches beneficial bacterial populations in gestating sows in a dose-dependent manner. Using an in vitro fermentation approach, this study systematically evaluated the effects of different AFM supplementation levels on the microbial composition and metabolomic profiles of fecal samples from gestating sows. The results will help elucidate the scientific and rational use of alfalfa fiber in gestating sow diets by providing theoretical support and will also establish a methodological reference for applying in vitro fermentation techniques in studies of the porcine gut microbiota, thereby promoting optimized feed utilization and improved production efficiency.

## 2. Materials and Methods

### 2.1. Test Materials

#### 2.1.1. Sample Preparation

AFM was derived from alfalfa (*Medicago sativa* L.) cultivated at the Xinxiang Yuanyang Experimental Base of Henan Agricultural University. Fresh plants were collected and separated into stems and leaves for morphological and nutritional composition analyses. Subsequently, the stems were thoroughly dried at 80 °C under temperature-controlled, light-protected conditions to constant weight, then ground and passed through a 1.00 mm sieve to obtain AFM [10].

#### 2.1.2. Preparation of Culture Medium

The fermentation medium was prepared according to the method of [11]. After modification, each liter of medium contained: soy peptone 13.00 g (Guangdong Huankai Microbial Technology Co., Ltd., Guangzhou, China), yeast extract 4.50 g (Angel Yeast Co., Ltd., Yichang, China), mucin 4.00 g (Yuanye Bio-Technology (Shanghai) Co., Ltd., Shanghai, China), L-cysteine HCl monohydrate 0.80 g (Yuanye Bio-Technology (Shanghai) Co., Ltd., Shanghai, China), bile extract (porcine) 0.40 g (Yuanye Bio-Technology (Shanghai) Co., Ltd., Shanghai, China), KH_2_PO_4_ 0.05 g (Sinopharm Chemical Reagent Co., Ltd., Shanghai, China), NaHCO_3_ 1.50 g (Sinopharm Chemical Reagent Co., Ltd., Shanghai, China), NaCl 4.50 g (Sinopharm Chemical Reagent Co., Ltd., Shanghai, China), KCl 4.50 g (Sinopharm Chemical Reagent Co., Ltd., Shanghai, China), MgSO_4_ (120.37 g/mol) 0.64 g (Sinopharm Chemical Reagent Co., Ltd., Shanghai, China), CaCl_2_·2H_2_O (147.02 g/mol) 0.15 g (Sinopharm Chemical Reagent Co., Ltd., Shanghai, China), MnCl_2_·4H_2_O (197.91 g/mol) 0.20 g (Sinopharm Chemical Reagent Co., Ltd., Shanghai, China), hemin solution (0.05 g/mL) 0.05 g and Tween 80 1.00 g (Yuanye Bio-Technology (Shanghai) Co., Ltd., Shanghai, China). Under high-temperature heating conditions, N_2_ (Sinopharm Chemical Reagent Co., Ltd., Shanghai, China) was simultaneously introduced through a flow divider, the medium pH (Shanghai Leici Instrument Factory (INESA Scientific/Leici), Shanghai, China) was adjusted to 6.8, and the prepared medium was dispensed into Celine vials (Jiangsu Huaou Glass Co., Ltd. (Huaou Glass), Yancheng, China) containing different substrates, 5 mL per vial. After sealing with stoppers, the vials were sterilized at 115 °C for 30 min (Shanghai Jinghong Laboratory Equipment Co., Ltd. (Jinghong), Shanghai, China).

#### 2.1.3. Animal and Fecal Sample Collection

In this experiment, four healthy multiparous crossbred DLY (Duroc × Landrace × Yorkshire) sows were selected, aged 80–130 weeks and weighing 210–240 kg, with similar parity and at 90–110 days of gestation. The feed formulation was as follows (Table 1). All experimental procedures and animal protection protocols were approved by the Animal Ethics Committee of Henan Agricultural University (Approval No: HENAU-2022-032). Fresh fecal samples (50 g) were collected from each pregnant sow using the colonic sampling method, and a 10% fecal suspension was prepared under anaerobic conditions in an anaerobic chamber using sterile phosphate-buffered saline (PBS) [12]. The diluted mixture was homogenized for 5 min using a homogenizer and filtered through three layers of sterile gauze to obtain the inoculum. Under anaerobic conditions, 500 μL of the inoculum was inoculated into Celine bottles containing substrate (0 mg, 50 mg, 100 mg, or 200 mg AFM) and 5 mL of culture medium using disposable sterile syringes. All procedures were performed under anaerobic conditions.

#### 2.1.4. Experimental Design

**Figure 1:**
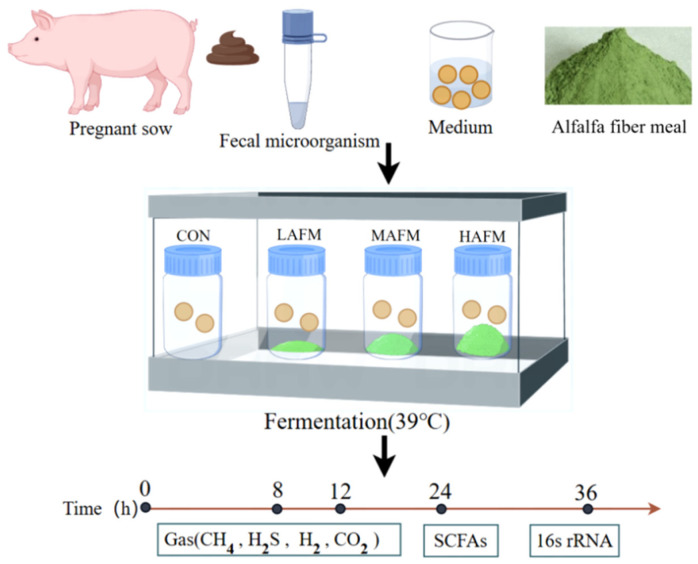
Study design roadmap.

The in vitro fermentation experiment was conducted using a batch culture method to simulate the conditions of the pig colon. Fresh feces from pregnant sows were collected and processed as inoculum. The experiment was carried out under sterile anaerobic conditions and divided into four groups: CON (0 mg), LAFM (50 mg), MAFM (100 mg), and HAFM (200 mg), with four replicates per group, as shown in Figure 1. In vitro fermentation was carried out by placing serum bottles (randomly assigned) containing substrate, culture medium, and inoculum in a constant-temperature incubator and fermenting them for 8, 12, 24, and 36 h at 39 ± 0.5 °C, respectively. Fermentation gas was measured using a gas analyzer; the supernatant was used for the determination of SCFAs, and the precipitate was analyzed by 16S rRNA sequencing.

#### 2.1.5. Measurement Indicators and Methods Determination of Gas Production Volume

At the end of fermentation, the fermentation bottles are brought to room temperature, and a gas analyzer is used for automatic gas analysis. The system was calibrated before and then used to determine gas composition and concentration. Gas composition analysis was performed using the Shanchuan series disc-type online gas component analyzer (AZSC series, former model APEF-H) manufactured by Shenzhen Ampere Technology Co., Ltd. (Shenzhen, China). The instrument consists of a gas pump and a detection module and is serviced periodically. The detection module uses electrochemical sensors to measure H_2_ and H_2_S, and infrared sensors to measure CO_2_ and CH_4_.

#### 2.1.6. Determination of Short-Chain Fatty Acids (SCFAs)

This study adopted and improved a method for determining SCFAs [13]. Fermentation supernatant (500 µL) was thoroughly mixed with a crotonic acid/methylphosphonic acid solution (100 µL) and stored at −40 °C for 24 h to allow acidification (deproteinization). Subsequently, 150 µL of the supernatant was transferred into an injection vial using a pipette, and the SCFAs in the culture were analyzed using a gas chromatograph (GC-2010 Plus, Shimadzu, Kyoto, Japan) equipped with a DB-FFAP capillary column (0.32 mm × 30 m × 0.5 μm, Agilent Technologies, Santa Clara, CA, USA) and a flame ionization detector.

#### 2.1.7. Determination of Microbial Composition in Fermentation Broth

The bacterial 16S rRNA gene V3–V4 hypervariable region was amplified using primers 338F (5′-ACTCCTACGGAGGCAGCAG-3′) and 806R (5′-GGACTACHVGGGTWTCTAAT-3′). The amplification products were recovered by 2% agarose gel electrophoresis and purified using the AxyPrep DNA Gel Recovery Kit (Axygen Biosciences, Union City, CA, USA). When the sample purity met the standard requirements, sequencing was performed on the Illumina MiSeq platform following standard operating procedures. Sequences with 97% similarity were clustered into operational taxonomic units (OTUs) through cluster analysis, and species classification information was obtained by comparison against the Silva 138 database. In addition, genomic analysis software (Visual Genomics Soft_V1.0.8x64) was used to perform analyses, including α-diversity, β-diversity, LEfSe analysis, and correlation analysis on the sequencing data.

### 2.2. Data Statistics and Analysis

After processing the raw data in Office, a one-way analysis of variance (ANOVA) was performed for each dataset using SPSS 26. Multiple comparisons were conducted using Duncan’s test. The results are presented as mean ± standard error of the mean (SEM). Differences were considered significant at *p* < 0.05 and highly significant at *p* < 0.01.

## 3. Results

### 3.1. This In Vitro Fermentation Gas Kinetics

In vitro fermentation demonstrated that the inclusion of AFM significantly influenced gas production characteristics (Table 2). Total gas and hydrogen production increased significantly (*p* < 0.05) with both increasing AFM inclusion level and fermentation time. Compared with the CON group, all AFM-supplemented groups exhibited significantly higher total gas and hydrogen yields at 8 h, 12 h, and 24 h. At 36 h, MAFM and HAFM maintained significantly elevated production (*p* < 0.05), with the HAFM group yielding the highest values. Methane production was significantly greater (*p* < 0.05) in all AFM groups than in the CON group during the early fermentation stages (8 h and 12 h). However, in the later stages (24 h and 36 h), only the HAFM group showed a significant increase in methane output. In contrast, hydrogen sulphide production was significantly reduced (*p* < 0.05) in all AFM groups throughout the entire fermentation period. Carbon dioxide production was significantly elevated (*p* < 0.05) solely in the HAFM group during the early phase (8 h and 12 h). However, by 24 h and 36 h, all AFM groups produced significantly more carbon dioxide than the CON group (*p* < 0.05).

### 3.2. Short-Chain Fatty Acid Profiles

In vitro fermentation results indicated that the inclusion of AFM significantly promoted the production of SCFAs. The total SCFA concentration increased significantly (*p* < 0.05) with increasing AFM inclusion levels at all fermentation time points and progressed over time (Table 3). Specifically, the yields of acetic, propionic, and butyric acids were significantly increased (*p* < 0.05) in the AFM-supplemented groups compared to the CON group at most time points. Butyric acid production did not differ significantly at 12 h but showed a significant dose-dependent effect at the other time points. Furthermore, the production of branched-chain fatty acids (isobutyric and isovaleric acids) was also significantly elevated (*p* < 0.05) during the mid-to-late fermentation stages (24 h and 36 h) in response to AFM supplementation, with the most pronounced effects observed in the HAFM. The pattern for valeric acid differed; its concentration was not affected by AFM during the early fermentation phases (8 h and 12 h), but became significantly higher (*p* < 0.05) in the AFM groups than in the CON group at 24 h and 36 h.

### 3.3. Microbial Diversity Patterns

**Figure 2:**
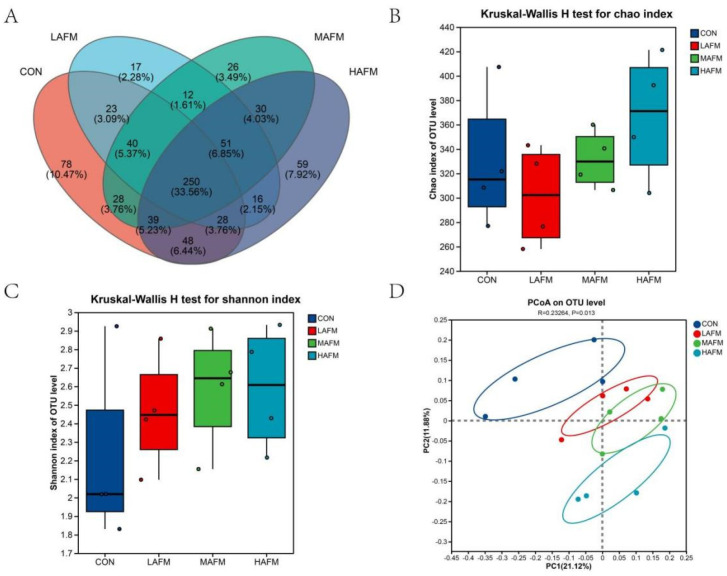
(**A**) Venn diagram, (**B**,**C**) α-diversity, and (**D**) β-diversity. CON was the control group; no AFM was added. LAFM, MAFM, and HAFM were the experimental groups, and the supplemental amounts of AFM were 50 mg, 100 mg, and 200 mg, respectively. The same as below.

Sequencing analysis of the in vitro fermentation microbiota was conducted to evaluate the effects of different levels of AFM on microbial community composition and diversity. Venn analysis at the OTU level revealed that the CON, LAFM, MAFM, and HAFM groups contained 17, 23, 26, and 12 unique OTUs, respectively (Figure 2A). A total of 40 OTUs were shared across all groups, indicating a core microbiota common to all treatments alongside distinct microbial constituents associated with each dietary intervention.

Further analysis of microbial α-diversity indicated that AFM supplementation did not significantly alter microbial richness or evenness. As shown in panels B–D of the figure below, no statistically significant differences were observed among treatment groups in either the sobs index or the Shannon index (Figure 2B,C). These results suggest that, under the experimental conditions used, the addition of AFM did not markedly affect the α-diversity of fecal microbial communities during in vitro fermentation inoculated with feces from pregnant sows.

Principal coordinates analysis (PCoA) based on Bray–Curtis distances revealed a significant divergence in microbial community structure between the CON group and the AFM-supplemented groups (R = 0.2326, *p* = 0.013, Figure 2D). The CON samples formed a distinct cluster separate from the LAFM, MAFM, and HAFM samples along the principal coordinates. The first two principal coordinates accounted for 11.87% and 21.12% of the total variation, respectively. This clear separation indicates that dietary AFM inclusion significantly altered the overall composition of the microbiota during in vitro fermentation, despite the absence of significant changes in alpha diversity.

### 3.4. Phylum-Level and Genus-Level Taxa Community Structure

**Figure 3:**
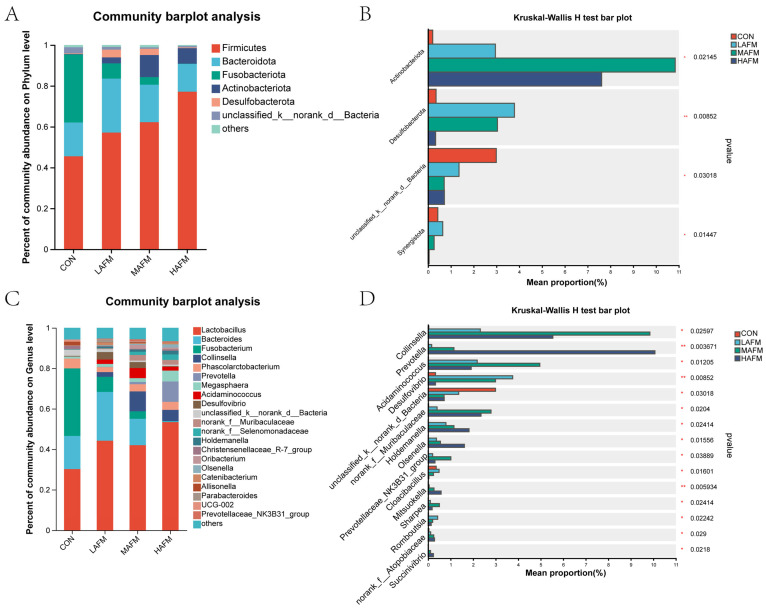
(**A**) Phylum-level composition of fermentation microbial communities. (**B**) Differential microorganisms at the phylum level of fermentation microbial communities. (**C**) Genus-level composition of fermentation microbial communities. (**D**) Differential microorganisms at the genus level of fermentation microbial communities. Statistical significance was defined as a *p* value < 0.05, with * *p* < 0.05 indicating a significant difference and ** *p* < 0.01 indicating a highly significant difference.

At the phylum level (Figure 3A), the fecal microbial communities across all experimental groups were primarily constituted by Firmicutes, Bacteroidota, and Fusobacteriota. A dose-responsive shift was observed in the relative abundance of Firmicutes, which increased progressively from 45.50% in the CON group to 77.14% in the HAFM group. Conversely, the abundance of Fusobacteriota demonstrated a substantial decline from 33.32% in the CON group to below 7.45% in all AFM-treated groups. Additionally, Desulfobacterota and Actinobacteria were present in the microbiota of AFM-supplemented groups but were virtually undetectable in the CON group.

Statistical comparisons at the phylum level revealed several significant inter-group differences (Figure 3B). The HAFM group exhibited a significantly greater abundance (*p* < 0.05) of Actinobacteria compared to the other three groups. Both LAFM and MAFM groups harbored significantly higher proportions (*p* < 0.05) of Desulfobacterota relative to the CON and HAFM groups. All AFM supplementation groups showed a marked reduction (*p* < 0.05) in unclassified bacterial taxa compared to the CON group. The abundance of Chloroflexi displayed a complex pattern, being significantly elevated (*p* < 0.05) in the LAFM group relative to CON, but significantly suppressed (*p* < 0.05) in both the MAFM and HAFM groups.

At the genus level (Figure 3C), dietary AFM inclusion induced substantial compositional changes in the microbial community. The CON group microbiota was predominantly characterized by *Clostridium* (33.32%) and *Lactobacillus* (30.18%). In contrast, AFM supplementation resulted in a progressive enrichment of *Lactobacillus*, culminating in a relative abundance of 53.40% in the HAFM group. Concurrently, several other genera, including *Prevotella*, *Collinsella*, and *Megasphaera*, showed increased representation in the AFM-treated groups compared to the control.

Differential abundance analysis further identified specific taxa that were significantly altered by AFM supplementation (Figure 3D). Multiple genera, including *Collinsella*, *Prevotella*, and *Acidaminococcus*, among others, were significantly more abundant (*p* < 0.05) in all three AFM groups relative to CON. The abundance of *Desulfovibrio* was significantly higher (*p* < 0.05), specifically in the LAFM and MAFM groups, compared to both CON and HAFM. In contrast, unclassified bacteria were significantly more prevalent (*p* < 0.05) in the CON group than in any AFM group, while *Cloacibacillus* showed significantly greater abundance (*p* < 0.05) in CON compared to the MAFM and HAFM groups.

### 3.5. Identification of Microbial Biomarkers by LEfSe Analysis

**Figure 4:**
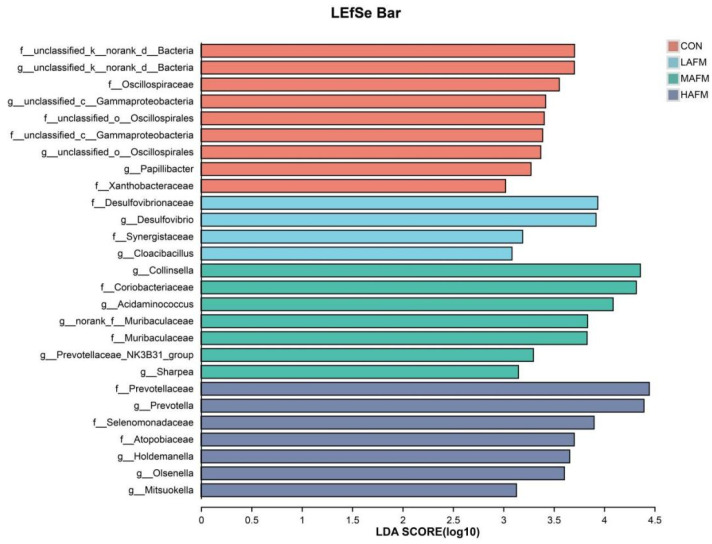
Linear discriminant analysis by LEfSe analysis (LDA > 3).

LEfSe analysis was performed to identify differentially abundant bacterial taxa across treatment groups, with a significance threshold of LDA score > 3.0. The analysis revealed distinct microbial biomarkers characteristic of each dietary regimen (Figure 4). The CON group was primarily defined by several unclassified taxa, including *g__unclassified_k__norank_d__Bacteria*, *g__unclassified_c__Gammaproteobacteria*, and *g__unclassified_o__Oscillospirales*, along with *g__Papillibacter*. In the LAFM group, *g__Desulfovibrio*, and *g__Cloacibacillus* emerged as significant biomarkers. The MAFM group exhibited a more complex biomarker profile, featuring *g__Collinsella*, *g__Acidaminococcus*, *g__norank_f__Muribaculaceae*, *g__Prevotellaceae_NK3B31_group*, and *g__Sharpea*. Notably, the HAFM group was characterized by a distinct consortium of biomarkers consisting of *g__Prevotella*, *g__Holdemanella*, *g__Olsenella*, and *g__Mitsuokella*. These results demonstrate that AFM supplementation induced dose-dependent alterations in the microbial community structure, with specific bacterial taxa serving as potential biomarkers for each treatment group. The progressive shift in microbial biomarkers from low to high AFM levels suggests a graded response of the gut microbiota to dietary fiber intervention, with increasing AFM concentrations selecting for distinct microbial populations of potential functional importance in fiber fermentation.

### 3.6. Correlation Analysis Between Microbial Genera and Fermentation Parameters

**Figure 5:**
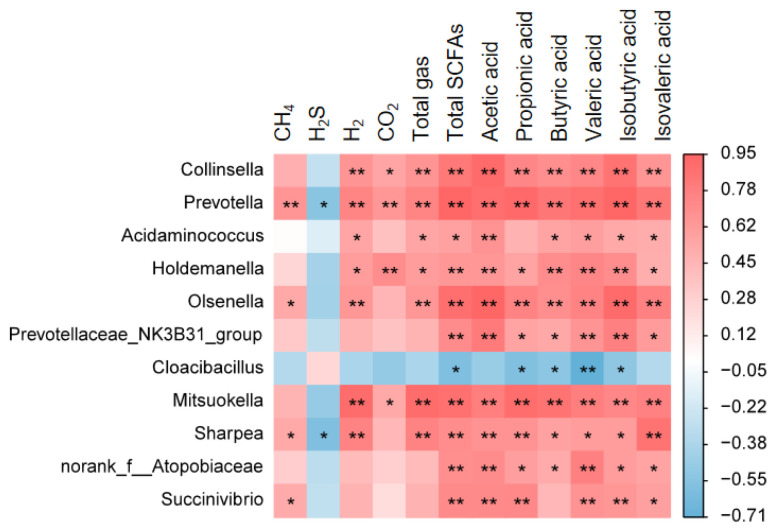
Correlation analysis of microorganisms with gases, SCFAs, and genus-level differences. Red represents positive correlation, blue represents negative correlation, and a darker color represents a stronger correlation, * indicates significant correlation (*p* < 0.05), ** indicates significant correlation (*p* < 0.01).

Correlation analysis was performed to elucidate the relationships between differentially abundant microbial genera and key fermentation parameters, including gas production and SCFAs concentrations (Figure 5). The results revealed significant associations between specific bacterial taxa and fermentation metabolites. Total SCFAs concentrations showed strong positive correlations with several genera, including *Collinsella*, *Prevotella*, *Holdemanella*, and *Olsenella* (*p* < 0.01), and a significant positive correlation with *Acidaminococcus* (*p* < 0.05). In contrast, total SCFAs were negatively correlated with *Cloacibacillus* abundance (*p* < 0.05). Gas production parameters also demonstrated significant microbial correlations. H_2_ production was strongly positively correlated with *Collinsella*, *Prevotella*, and *Olsenella* (*p* < 0.01), and significantly positively correlated with *Acidaminococcus* and *Holdemanella* (*p* < 0.05). Conversely, H_2_S production exhibited significant negative correlations with *Prevotella* and *Sharpea* (*p* < 0.05). These correlation patterns suggest that AFM-induced changes in specific microbial populations, particularly the enrichment of *Collinsella*, *Prevotella*, and *Olsenella*, are associated with enhanced carbohydrate fermentation capacity, as reflected by increased SCFAs production and hydrogen gas generation. The negative correlation between *Cloacibacillus* and total SCFAs further supports the role of AFM in shaping a microbial community structure optimized for fiber degradation.

## 4. Discussion

The present study systematically investigated the effects of graded levels of AFM on in vitro fermentation characteristics, microbial community structure, and metabolic profiles of microbiota derived from pregnant sows. Our results demonstrated that AFM supplementation induced dose-dependent modulations in gas production, SCFAs profiles, and microbial composition, revealing a coherent pattern of enhanced fiber fermentation coupled with microbial ecosystem optimization.

Gases produced during microbial metabolism of dietary fiber can serve as important indicators of colonic fermentation status and gut ecological dynamics [14], and they also participate in regulating intestinal physiological functions [15]. In this study, as AFM levels and fermentation time increased, total gas production as well as H_2_ and CO_2_ production increased overall, suggesting enhanced substrate accessibility and activation of carbohydrate fermentation–related pathways [16]. Because CO_2_ typically originates from carbohydrate breakdown and decarboxylation processes, whereas H_2_ mainly reflects the release and transfer of reducing equivalents during fermentation, a concurrent increase in both can be regarded as evidence of enhanced microbial energy metabolism and an increased overall community fermentation rate. Notably, in the early stage of fermentation, methane production increased significantly in all AFM groups, but this difference diminished over time. Methane formation depends on methanogenic archaea utilizing CO_2_ [17]; therefore, its early increase may indicate that AFM rapidly releases fermentable substrates at the initial stage, resulting in increased H_2_ production from fermentation and thereby providing sufficient substrates for methanogens and conferring a competitive advantage over a short period. These findings are consistent with previous research [18] and further suggest that AFM-driven community dynamics may undergo a process of “rapid early fermentation—stabilization in the mid-to-late stages.” More importantly, all AFM groups continuously suppressed H_2_S production. Because H_2_S is often associated with the degradation of sulfur-containing amino acids and sulfate reduction, its decrease is generally considered reflective of a shift in microbial metabolism from proteolysis-related pathways toward carbohydrate utilization [19]. At higher levels, H_2_S may impair mitochondrial function in the intestinal epithelium [20], weaken the mucosal barrier, and trigger inflammatory responses; therefore, the inhibitory effect of AFM on H_2_S suggests that it may improve the gut microenvironment by reducing protein fermentation and the accumulation of toxic metabolites. In addition, preferential fermentation of carbohydrates is usually associated with increased SCFAs production; these metabolites can lower luminal pH, inhibit certain opportunistic pathogens, and provide an energy source for colonic epithelial cells, thereby further promoting microecological homeostasis and barrier function [21]. Overall, this study collectively indicates that AFM can optimize the intestinal environment by providing fermentable substrates, selectively promoting potentially beneficial microbial communities, and suppressing potentially harmful metabolic processes.

In in vitro fermentation systems, SCFAs are among the most important and most frequently detected terminal metabolites [22]. They not only reflect the extent to which microorganisms utilize carbohydrates but also serve as key indicators for evaluating the functional output of the microbiota and are closely associated with probiotic proliferation and metabolic activity [23]. Consistent with changes in gas-production kinetics, AFM supplementation significantly promoted SCFA production in a dose- and time-dependent manner. Given that microbial fermentation is the primary source of SCFAs and that SCFAs are considered important mediators of gut health [24], the continuous accumulation of SCFAs can be regarded as direct evidence that AFM improves the fermentation environment and enhances functional metabolic output. The gradual increase in total SCFA concentration indicates that AFM effectively improved the efficiency of carbohydrate fermentation. This process is typically accompanied by luminal acidification and a decrease in pH, which at the ecological-niche level suppresses certain acid-sensitive potential pathogens while providing more favorable competitive conditions for acid-producing bacteria [25]. In addition, SCFAs are not only important energy substrates for the colonic epithelium but can also exert anti-inflammatory and immunomodulatory effects by regulating mucosal barrier integrity and influencing immune signaling pathways [26]. In terms of composition, acetate—the most abundant SCFA—was significantly higher in all AFM groups than in the control, suggesting that the dietary fiber in AFM can rapidly enter primary fermentation pathways and may provide a metabolic basis for the subsequent accumulation of high-carbon SCFAs [27]. Butyrate and propionate reached significant levels in the later stage of fermentation, and their concentrations were higher in all AFM-treated groups, indicating that optimal synthesis of different SCFAs often depends on the availability of specific substrates and the temporal succession of community structure [28]. Valerate production is generally considered to be associated with the further transformation of other SCFAs or secondary fermentation of specific substrates, possibly reflecting that fermentation has entered a deeper metabolic stage. Isobutyrate and isovalerate are mostly related to the fermentation of branched-chain amino acids and are often regarded as indicators of enhanced protein fermentation [29], which also explains why they increased significantly only at later time points.

This optimized fermentation metabolic pattern was closely associated with the reshaping of the microbial community structure. Although AFM did not alter the alpha diversity within samples, it significantly remodeled the microbial community composition [30]. Principal coordinate analysis revealed a clear separation between the CON and AFM groups, confirming AFM’s ability to modify the gut microbial community structure. At the phylum level, the proportion of Firmicutes increased in a dose-dependent manner, making it the dominant phylum; other in vivo studies in humans have reported similar findings and have shown that these microorganisms exert significant effects on host health, immune function, and metabolism [31]. The marked increase in Bacteroidetes suggested that this phylum, as a major producer of SCFAs, played an important role in maintaining gut health [32]. Meanwhile, Fusobacterium was closely associated with disease [33], and we found that a significant reduction in Fusobacterium abundance indicated the potential of AFM in suppressing harmful microbial populations. In addition, the increased abundance of actinomycetes further underscores AFM’s role in promoting a healthier microbial community structure, which is consistent with previous in vivo experimental results [34].

At the genus level, these phylum-level changes manifested as the enrichment or suppression of specific functional groups. A significant increase in lactic acid bacteria inhibited the growth of pathogenic bacteria through the production of lactic acid, thereby improving intestinal health [35]. Studies have reported a positive correlation between *Clostridium* species and gastrointestinal diseases [36], while our experiment found a significant reduction in *Clostridium* species, indicating that AFM promoted the growth of beneficial bacteria while inhibiting the proliferation of harmful bacteria. The elevated abundance of *Collinsella* [37], along with the enrichment of *Prevotella*, *Olsenella,* and *Holdemanella* [38,39,40], all showed a gradient increase with higher AFM levels, suggesting that the microbial community was adapting and specializing in dietary fiber degradation. The strong positive correlations of these genera with SCFA and hydrogen production further reinforced their central roles in the AFM fermentation process.

In summary, AFM dose-dependently improves fermentation by the gut microbiota in pregnant sows by promoting gas production and short-chain fatty acid (SCFA) generation, increasing the abundance of beneficial bacteria, and inhibiting harmful bacteria. Under the conditions of this experiment, 100 mg was identified as a more balanced supplemental level of this fermentation additive for pregnant sows, primarily applicable to pregnant sows; its applicability to other physiological stages still needs to be further verified in future studies.

## Figures and Tables

**Table 1:** Ingredients and nutrient levels of the gestation diet.

Items	Contents
Ingredients	
Corns/%	51.40
Soybean meal/%	18.40
Bran/%	7.50
Rice bran meal/%	4.00
Wheat flour/%	10.00
Fish meal/%	1.50
Soy phospholipid flour/%	1.00
Soybean oil/%	2.20
Premix/% *	4.00
Total	100.00
Calculated nutrition levels	
Metabolizable energy, MJ/kg	13.40
Net energy, MJ/kg	10.13
Crude protein/%	16.50
Crude fiber/%	3.50
Calcium/%	0.85
Total phosphorus/%	0.60
Lysine/%	1.05
Threonine/%	0.65

Note: * Premix supplied with following per kg complete diet: vitamin A, 8000 IU; vitamin D3, 2000 IU; vitamin E, 40 mg; vitamin K, 2 mg; vitamin B1, 3 mg IU; vitamin B2, 5 mg; vitamin B6, 3 mg; vitamin B12, 0.04 mg; nicotinic acid, 30 mg; vitamin B5, 20 mg; folic acid, 1.5 mg; biotin 0.3 mg; riboflavin 8 mg; Fe, 100 mg; Cu, 20 mg; Zn, 80 mg; Mn, 40 mg; I, 0.3 mg; Se, 0.25 mg.

**Table 2:** Effects of different levels of AFM on the gas content of fecal fermentation in pregnant sows.

Items	Groups				SEM	*p*-Value
	CON	LAFM	MAFM	HAFM		ANOVA
Total gas/mL						
8 h	12,267.18 ^b^	13,529.74 ^a^	14,119.18 ^a^	14,121.50 ^a^	228.628	*p* < 0.05
12 h	13,715.01 ^c^	15,125.58 ^b^	15,725.07 ^ab^	16,742.19 ^a^	296.055	*p* < 0.05
24 h	10,893.55 ^b^	12,534.90 ^a^	12,940.21 ^a^	12,936.65 ^a^	254.953	*p* < 0.05
36 h	10,422.96 ^c^	11,289.47 ^bc^	12,214.38 ^ab^	12,716.66 ^a^	255.811	*p* < 0.05
H_2_/mL						
8 h	12,061.63 ^b^	13,338.8 ^a^	13,931.64 ^a^	13,931.61 ^a^	230.152	*p* < 0.05
12 h	13,514.20 ^c^	14,898.65 ^b^	15,482.41 ^ab^	16,482.41 ^a^	291.706	*p* < 0.05
24 h	10,685.61 ^b^	12,336.23 ^a^	12,739.86 ^a^	12,739.83 ^a^	255.878	*p* < 0.05
36 h	10,222.43 ^c^	11,097.21 ^bc^	12,018.09 ^ab^	12,518.05 ^a^	255.686	*p* < 0.05
CH_4_/mL						
8 h	92.21 ^b^	105.23 ^a^	111.21 ^a^	116.89 ^a^	2.757	*p* < 0.05
12 h	99.61 ^b^	112.62 ^a^	115.43 ^a^	123.67 ^a^	2.630	*p* < 0.05
24 h	122.09 ^b^	126.25 ^ab^	131.61 ^ab^	134.89 ^a^	2.029	*p* > 0.05
36 h	125.25 ^b^	128.03 ^ab^	133.65 ^ab^	138.24 ^a^	1.976	*p* > 0.05
H_2_S/mL						
8 h	112.46 ^a^	84.64 ^b^	75.29 ^c^	71.85 ^c^	3.927	*p* < 0.05
12 h	102.41 ^a^	81.21 ^b^	71.46 b^c^	66.63 ^c^	3.531	*p* < 0.05
24 h	83.89 ^a^	70.28 ^b^	66.49 ^bc^	58.85 ^c^	2.525	*p* < 0.05
36 h	73.04 ^a^	61.84 ^b^	60.27 ^b^	57.22 ^b^	1.926	*p* < 0.05
CO_2_/mL						
8 h	0.98 ^b^	1.14 ^ab^	1.18 ^ab^	1.30 ^a^	0.045	*p* > 0.05
12 h	1.61 ^b^	1.78 ^b^	1.87 ^b^	2.19 ^a^	0.063	*p* < 0.05
24 h	1.76 ^c^	2.15 ^b^	2.30 ^ab^	2.41 ^a^	0.066	*p* < 0.05
36 h	2.02 ^b^	2.36 ^a^	2.47 ^a^	2.58 ^a^	0.060	*p* < 0.05

No letter or the same letter in peer data shoulder indicates no significant difference (*p* > 0.05), while different lowercase letters indicate a significant difference (*p* < 0.05). The same as below.

**Table 3:** Effects of different levels of AFM on short-chain fatty acid content during in vitro fermentation of feces from pregnant sows.

Items	Groups				SEM	*p*-Value
	CON	LAFM	MAFM	HAFM		ANOVA
Total SCFAs/(mmol/L)						
8 h	9.47 ^c^	12.40 ^b^	13.78 ^ab^	15.36 ^a^	0.604	*p* < 0.05
12 h	20.43 ^c^	25.46 ^b^	27.64 ^a^	29.69 ^a^	0.851	*p* < 0.05
24 h	31.49 ^c^	44.23 ^b^	46.84 ^ab^	50.05 ^a^	1.690	*p* < 0.05
36 h	41.73 ^d^	55.53 ^c^	60.68 ^b^	65.98 ^a^	2.113	*p* < 0.05
Acetic acid/(mmol/L)						
8 h	6.95 ^b^	6.95 ^a^	9.76 ^a^	10.46 ^a^	0.404	*p* < 0.05
12 h	7.42 ^b^	11.13 ^a^	11.66 ^a^	12.50 ^a^	0.523	*p* < 0.05
24 h	13.38 ^c^	18.06 ^b^	19.35 ^ab^	20.71 ^a^	0.690	*p* < 0.05
36 h	17.15 ^c^	22.11 ^b^	23.23 ^ab^	24.81 ^a^	0.706	*p* < 0.05
Propionic acid/(mmol/L)						
8 h	1.47 ^b^	1.74 ^ab^	2.06 ^ab^	2.37 ^a^	0.124	*p* < 0.05
12 h	2.56 ^b^	2.74 ^b^	3.39 ^ab^	3.81 ^a^	0.174	*p* < 0.05
24 h	7.19 ^b^	8.81 ^a^	9.08 ^a^	9.41 ^a^	0.254	*p* < 0.05
36 h	10.37 ^d^	11.48 ^c^	13.77 ^b^	15.12 ^a^	0.453	*p* < 0.05
Butyric acid/(mmol/L)						
8 h	1.33 ^c^	1.33 ^bc^	1.87 ^b^	2.43 ^a^	0.154	*p* < 0.05
12 h	4.41	4.58	4.79	5.06	0.115	*p* > 0.05
24 h	5.34 ^b^	7.63 ^a^	7.30 ^a^	7.81 ^a^	0.264	*p* < 0.05
36 h	5.75 ^c^	7.86 ^b^	8.43 ^ab^	9.15 ^a^	0.315	*p* < 0.05
Valeric acid/(mmol/L)						
8 h	0.02	0.02	0.02	0.02	0.001	*p* > 0.05
12 h	0.02	0.02	0.02	0.02	0.001	*p* > 0.05
24 h	2.43 ^c^	4.63 ^b^	5.54 ^b^	6.13 ^a^	0.344	*p* < 0.05
36 h	3.97 ^c^	6.82 ^b^	7.43 ^b^	8.49 ^a^	0.399	*p* < 0.05
Isobutyric acid/(mmol/L)						
8 h	0.02	0.02	0.02	0.02	0.001	*p* > 0.05
12 h	0.02 ^c^	0.02 ^bc^	0.03 ^ab^	0.03 ^a^	0.001	*p* < 0.05
24 h	1.11 ^b^	1.47 ^a^	1.56 ^a^	1.56 ^a^	0.064	*p* < 0.05
36 h	1.47 ^c^	1.81 ^b^	2.02 ^b^	2.43 ^a^	0.092	*p* < 0.05
Isovaleric acid/(mmol/L)						
8 h	0.05	0.05	0.05	0.05	0.001	*p* > 0.05
12 h	0.04 ^b^	0.05 ^b^	0.05 ^b^	0.06 ^a^	0.002	*p* < 0.05
24 h	2.04 ^c^	3.65 ^b^	4.02 ^b^	4.49 ^a^	0.221	*p* < 0.05
36 h	3.03 ^b^	5.47 ^a^	5.83 ^a^	6.00 ^a^	0.287	*p* < 0.05

No letter or the same letter in peer data shoulder indicates no significant difference (*p* > 0.05), while different lowercase letters indicate a significant difference (*p* < 0.05).

## Data Availability

The original data presented in the study are openly available in the Sequence Read Archive (SRA) of the China National Center for Bioinformation (CNCB) under the accession number PRJNA1380028.

## Abbreviations

The following abbreviations are used in this manuscript:

AFM	alfalfa fiber meal
SCFAs	short-chain fatty acid
FMT	fecal microbiota transplantation
